# TGF-*α* Overexpression in Breast Cancer Bone Metastasis and Primary Lesions and TGF-*α* Enhancement of Expression of Procancer Metastasis Cytokines in Bone Marrow Mesenchymal Stem Cells

**DOI:** 10.1155/2018/6565393

**Published:** 2018-02-08

**Authors:** Jingbo Sun, Haiyan Cui, Yanxin Gao, Yangjian Pan, Kun Zhou, Jingzhan Huang, Jin Lan, Qingzhu Wei, Xiaolong Liu, Lixin Liu, Cory J. Xian

**Affiliations:** ^1^Department of General Surgery, The Third Affiliated Hospital of Southern Medical University, Guangzhou, Guangdong 510630, China; ^2^Department of Internal Medicine, The Third Affiliated Hospital of Southern Medical University, Guangzhou, Guangdong 510630, China; ^3^Department of Pathology, The Third Affiliated Hospital of Southern Medical University, Guangzhou, Guangdong 510630, China; ^4^Sansom Institute for Health Research, School of Pharmacy and Medical Sciences, University of South Australia, Adelaide, SA 5001, Australia

## Abstract

Bone metastasis (BM) is the advanced complication of breast cancer, while bone marrow-derived mesenchymal stem cells (BMSCs) in the microenvironment unclearly contribute to cancer metastasis. This study investigated potential roles of transforming growth factor- (TGF-) *α* in the interaction between breast cancer and BMSCs in BM. Clinical cases of breast cancer with bone metastasis (BMBC), breast cancer without bone metastasis (Non-BM-BC), and benign fibroadenoma (Benign) were enlisted in a retrospective study. TGF-*α* was found obviously overexpressed in BM lesion of BMBC compared to primary lesion of both BMBC and Non-BM-BC (*P* < 0.01), and TGF-*α* was higher in primary lesion of both BMBC and Non-BM-BC (*P* < 0.01) than Benign group. Interestingly, TGF-*α* in nontumor tissues of both BMBC and Non-BM-BC was at a higher level than Benign group (*P* < 0.01), and numbers of macrophages in nontumor tissues of both BMBC and Non-BM-BC (*P* < 0.01) were higher than Benign group. Furthermore, in cultured human BMSCs, TGF-*α* stimulated production of procancer cytokines including IL-6, VEGF, FGF10, FGF17, and TGF-*β*1 in a dose-dependent manner. Thus, TGF-*α* in BC could potentially be an important signal of carcinogenesis and metastasis. Macrophages in the nontumor tissue of BC may not be protective but could promote cancer metastasis.

## 1. Introduction

Breast cancer (BC) is the number 1 malignant tumor among females worldwide, and malignant metastasis is the major cause of breast cancer related death. About 70% BC cases are known to develop bone metastasis, and even 20% BC cases have bone metastasis in the early stage of primary lesion [1, 2]. Bone metastasis breast cancer (BMBC) is a complicated and advanced stage that starts in the primary tumor microenvironment. Tumor microenvironment is composed of tumor cells, mesenchymal stem cells (MSCs), fibroblasts, immune cells, vascular endothelial cells, other stromal cells, and extracellular matrix [3]. It has been accepted that the interaction between BC cells and components in the microenvironment could influence the progression of BC [4].

MSCs are multipotent stem cells that are mainly derived from bone marrow, fat tissue, umbilical cord blood, and dental pulp [5–7]. Bone marrow-derived MSCs (BMSCs) are progenitors of fibroblasts, adipocytes, osteoblasts, and cartilage cells in normal human microenvironment and play important roles in tissue regeneration [8, 9]. In the tumor microenvironment, BMSCs produce cytokines and exosomes to promote tumor invasion and metastasis. It has been shown that plasminogen activator inhibitor- (PAI-) 1, interleukin- (IL-) 6, Notch 1, and CD44 produced from BMSCs improve colorectal cancer cell survival and promote its development [10]. IL-6 and vascular endothelial growth factor (VEGF) from BMSCs improve BC metastasis, and the phenomenon was enhanced when BC cells are exposed to IL-6 and VEGF together [11]. In three-dimensional culture, BC cells developed invasion capacity when treated with transforming growth factor- (TGF-) *β*1 from BMSCs [12]. BC cells have been reported to be able to capture and cannibalize BMSCs in the microenvironment which can enable them to change into a dormant state that can increase drug resistance and immunosuppression and finally increase cancer recurrence [13].

Transforming growth factor- (TGF-) *α* was reported by bioinformatic analyses to potentially stimulate BMSCs to promote breast cancer [14]. TGF-*α* is a transforming growth factor as part of a human 160-amino-acid transmembrane precursor, and it is a ligand for the epidermal growth factor receptor which plays roles in tissue regeneration and bone homeostasis and may promote tumorigenesis [15–17]. Elevated TGF-*α* is associated with the tumorigenesis of the breast and stomach [18, 19]. However, whether TGF-*α* has a role in stimulating BMSCs during breast cancer bone metastasis still remains unclear.

In this study, as steps for identifying the role of TGF-*α* in breast cancer bone metastasis, expression of TGF-*α* in primary lesion and bone metastasis of breast cancer was analyzed and the influence of TGF-*α* on BMSCs was evaluated.

## 2. Method and Materials

### 2.1. Human Cases

Informed consent from all the patients was obtained and the study was approved by Ethics Board of the Third Affiliated Hospital of Southern Medical University.

#### 2.1.1. Breast Cancer with Bone Metastasis (BMBC) Group

**Figure 1:**
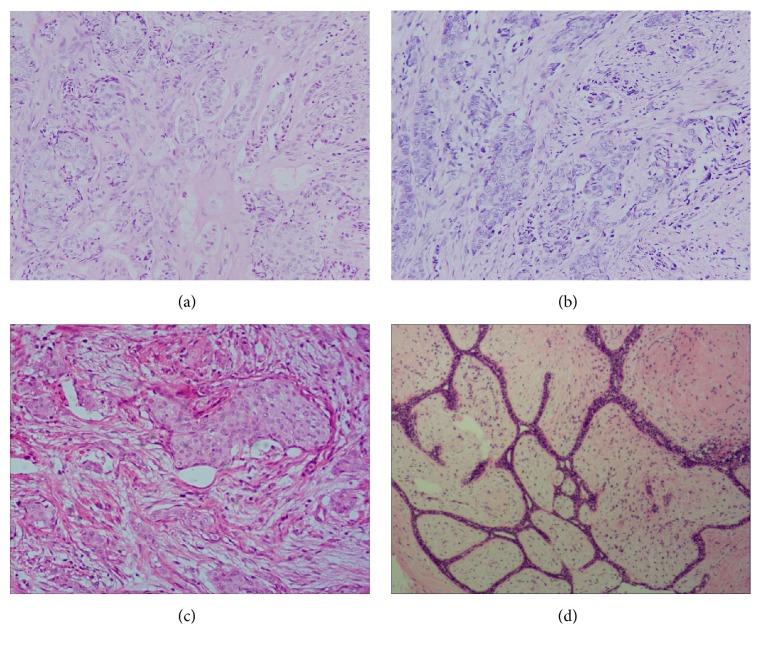
Histopathological diagnosis (H&E staining) of samples from the 3 groups. (a) Breast cancer bone metastasis (200x). (b) Primary lesion of breast cancer with bone metastasis (200x), showing nonspecific invasive ductal carcinoma, WHO grade III. (c) Primary lesion of breast cancer without bone metastasis (200x), showing nonspecific invasive ductal carcinoma, WHO grade III. (d) Breast fibroadenoma (200x).

Four female breast cancer cases with bone metastasis were enrolled in the retrospective study as the observation group. The 4 cases were diagnosed with breast cancer bone metastasis and received both mastectomy and excision of bone metastasis. The rule-in criteria also included no involvements of central nervous system diseases, gynecological diseases, or autoimmune diseases that might influence breast cancer progression and TGF-*α* expression. Their primary lesions were in the early stage of the TNM staging system [20]. The clinical data of these 4 BMBC cases were listed in Table 1 and shown in Figure 1.

#### 2.1.2. Breast Cancer without Bone Metastasis (Non-BM-BC) Control Group

Four female breast cancer cases without bone metastasis were enrolled in the study as Non-BM-BC control group. The 4 cases were diagnosed with resectable breast cancer without bone metastasis (Non-BM-BC) and received mastectomy. The rule-in criteria included age being above 40 years to balance the influence of age, specific pathological diagnosis results (nonspecific invasive ductal carcinoma, WHO grade III; primary lesion, T2; and molecular group: luminal B), and no involvements of central nervous system diseases, gynecological diseases, or autoimmune diseases that might influence breast cancer progression and TGF-*α* expression. Their primary lesions were in the early stage of TNM staging.

#### 2.1.3. Benign Control Group

Four female breast fibroadenoma cases were enlisted in the study as the negative control group. The 4 cases were diagnosed with potentially malignant breast lump before operation and received lumpectomy. The postoperative pathological results showed breast fibroadenoma. The rule-in criteria included age above 40 years and no involvements of central nervous system diseases, gynecological diseases, or autoimmune diseases. Their primary lesions were in the early stage of TNM staging.

### 2.2. Immunohistochemical Analyses (IHC)

Samples of primary lesions and bone metastasis were fixed in formalin and embedded in paraffin and analyzed by immunohistochemical analysis. The 4 *μ*m sections were deparaffinized and rehydrated, and their endogenous peroxidase activity was inhibited with 0.3% H_2_O_2_ methanol. After being blocked with the 5% normal goat serum for 1 hour at room temperature, the slides were incubated with the following primary antibodies at 4°C overnight: anti-TGF-*α* polyclonal antibody (ImmunoWay, Plano, TX, 1 : 50), anti-CD68 antibody (Abcam, Cambridge, UK; 1 : 200), anti-IL-6 antibody (ImmunoWay, Plano, TX, 1 : 50), anti-VEGF antibody (ImmunoWay, Plano, TX, 1 : 100), anti-FGF10 antibody (ABclonal, Boston, USA, 1 : 100), anti-FGF17 antibody (ImmunoWay, Plano, TX, 1 : 100), and anti-TGF-*β*1 antibody (ABclonal, Boston, USA, 1 : 100). Following incubation with biotinylated secondary antibodies, the streptavidin-biotin complex/horseradish peroxidase was applied. Finally, the immunoreaction signal was developed with DAB staining, and the slides were counterstained in hematoxylin.

The stained tissue sections were reviewed under a light microscope (Nikon ECLIPSE Ni-U, Tokyo, Japan). The numbers of TGF-*α* and CD68 positive cells were counted in 5 random fields (400x) of paraneoplastic tissue of each tissue section, respectively.

### 2.3. Cell Culture

Human bone marrow-derived MSCs (hBMSCs) were purchased from Guangzhou Jenniobio Biotechnology Co., Ltd (Guangzhou, China), and cultured in DMEM medium (GIBCO, Gaithersburg, MD) supplemented with 10% fetal bovine serum (HyClone, Logan, UT) and 1% penicillin/streptomycin (Invitrogen, Waltham, MA) in a 5% CO_2_ humidified atmosphere at 37°C. TGF-*α* exposure experiments were performed using hBMSCs between passages three and seven. Human BMSCs were treated for 8 or 24 hours with recombinant human TGF-*α* (Sino Biological, Beijing, China) at a concentration of 0 (medium control), 10, or 20 ng/ml.

### 2.4. Quantitative PCR (q-PCR)

Total RNA was extracted from untreated or TGF*α*-treated hBMSCs using Trizol reagent (Invitrogen), and cDNA was synthesized using an access RT system (Promega, Madison, WI). Real-time PCR was performed using ABI7500 Real-Time PCR System and SYBR Premix ExTaq II kit (TaKaRa Biotechnology Co., Ltd, Dalian, China). The primer sequences were selected as follows: IL-6, forward primer CATCCTCGACGGCATCTCAG and reverse primer ACCAGGCAAGTCTCCTCATTG; VEGF, forward primer CATCACCATGCAGATTATGCGG and reverse primer GAGGCTCCAGGGCATTAGAC; fibroblast growth factor (FGF) 10, forward primer CCGTACAGCATCCTGGAGATAAC and reverse primer CCTCCCATTATGCTGCCAGTT; FGF 17, forward primer CCCAACCTCACTCTGTGCTTAC and reverse primer TGTAGAGTTGGTACTCGCGG; TGF-*β*1, forward primer CGACTCGCCAGAGTGGTTAT and reverse primer GGTAGTGAACCCTGCGTTGAT. The PCR condition was 95°C for 30 s, followed by 40 cycles of amplification (95°C for 5 s, 60°C for 34 s, and 72°C for 34 s). The relative quantification was determined using the ΔΔ^−Ct^ method and mRNA expression levels were normalized to internal control gene GAPDH. Each sample was tested three times.

### 2.5. Statistics

SPSS 19.0 (IBM SPSS Inc., Chicago, IL) was used to evaluate data. One-way analysis of variance followed by LSD *t*-test was used to analyze differences between groups, and 2-tailed significance was determined. Results are presented as the mean ± standard deviation (SD) for all parameters measured. *P* < 0.05 was considered statistically significant.

## 3. Results

### 3.1. TGF-*α* in Bone Metastasis and Primary Lesion of Breast Cancer and Benign Control

**Figure 2:**
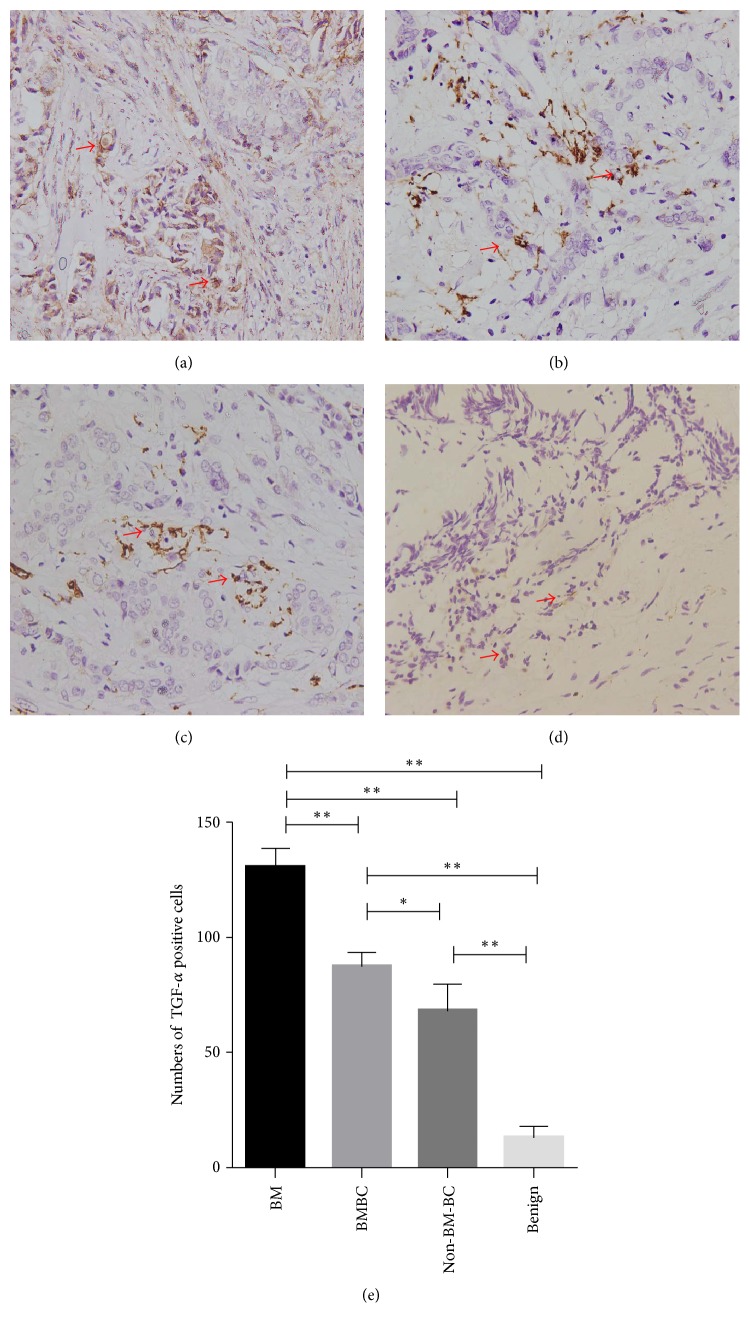
TGF-*α* in different tumor tissue. (a) TGF-*α* in bone metastasis (BM) lesion of breast cancer (400x), with arrows pointing to TGF-*α* positive cells; (b) TGF-*α* in primary lesion of breast cancer with bone metastasis (BMBC) (400x); (c) TGF-*α* in primary lesion of breast cancer without bone metastasis (Non-BM-BC) (400x); (d) TGF-*α* in benign breast fibroadenoma (400x); (e) comparison of TGF-*α* positive cells in tumor lesion of different groups (total positive cells in 5 random fields). ^*∗*^*P* < 0.05; ^*∗∗*^*P* < 0.01.

As analyzed by immunohistochemistry, bone metastases had a higher level TGF-*α* (more positive cells) than their own primary lesion (*P* < 0.01). TGF-*α* level in primary lesion of BMBC group was higher than that in primary lesion of BC without bone metastasis (*P* < 0.05) (Figure 2). Benign control had a much lower level of TGF-*α* than the bone metastasis (*P* < 0.01) and all primary lesions (*P* < 0.01) (Figure 2).

**Figure 3:**
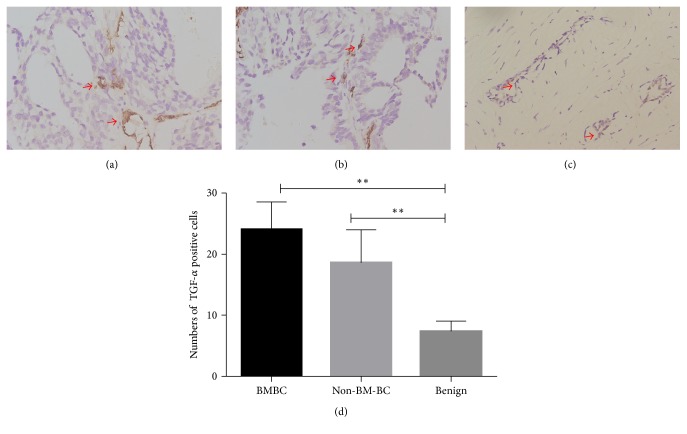
TGF-*α* expression in different nontumor tissues around tumor. (a) TGF-*α* in nontumor tissues around primary lesion of breast cancer with bone metastasis (BMBC) (400x, arrows pointing to TGF-*α* positive cells); (b) TGF-*α* in nontumor tissues around primary lesion of breast cancer without bone metastasis (Non-BM-BC) (400x); (c) TGF-*α* in nontumor tissues around benign breast fibroadenoma (400x); (d) comparison of TGF-*α* positive cells in nontumor tissues of different groups (total positive cells within 5 random fields). ^*∗∗*^*P* < 0.01.

Interestingly, TGF-*α* is expressed at higher levels in the nontumor tissues of both BMBC and Non-BM-BC while it was seldom found in nontumor tissues of the benign control (*P* < 0.01) (Figure 3). No significant differences were found in TGF-*α* levels in the nontumor tissues between BMBC and Non-BM-BC groups (*P* > 0.05) (Figure 3).

### 3.2. Macrophages (CD68+ Cells) in BC Lesion and Nontumor Tissues

**Figure 4:**
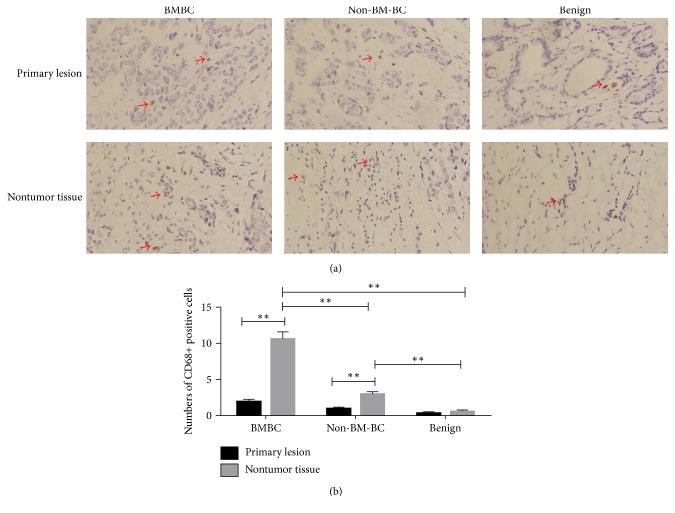
Numbers of macrophages (CD68+ cells) in different tissues. (a) Macrophages in primary lesion and nontumor tissues (400x, arrows indicating CD68+ positive cells); (b) comparison of macrophage numbers in different tissues. ^*∗∗*^*P* < 0.01.

Since macrophages are a known source of TGF-*β*1 in breast cancer, macrophages were detected by CD68 immunohistochemistry and positive cells were counted in BC lesion and nontumor tissues. Macrophages in BC lesion were found to be fewer than the nontumor tissue (*P* < 0.01) (Figure 4). Numbers of macrophages in BC lesion were not different from those in benign lesion. Numbers of macrophages in nontumor tissue of BMBC and Non-BM-BC were higher than those in nontumor tissue of benign control (*P* < 0.01).

### 3.3. Induction of Cytokines and Growth Factors from Human BMSCs under TGF-*α* Stimulation and Their Increased Expression Levels in Different Nontumor Tissues around Tumor

**Figure 5:**
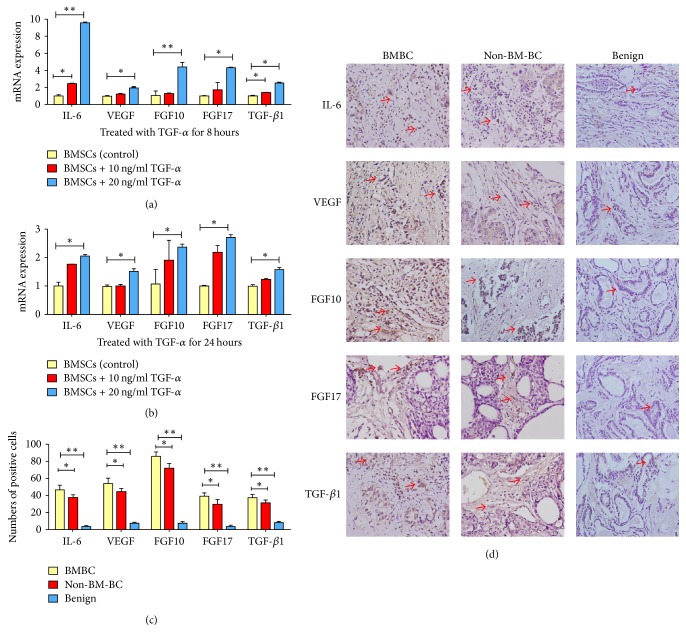
Changes in mRNA expression of cytokines and growth factors in BMSCs treated with different concentrations of TGF-*α*. (a) 8 hours after treatment; (b) 24 hours after treatment; (c) comparison of cytokines and growth factors expression in different nontumor tissues around tumor. ^*∗*^*P* < 0.05; ^*∗∗*^*P* < 0.01 compared with no treatment control; (d) cytokines and growth factors expression in different nontumor tissues around tumor (400x, arrows indicating positive cells).

Effects of TGF-*α* stimulation on expression of known genes involved in breast cancer metastasis were examined in human BMSCs. As analyzed by q-PCR, within 8 hours of TGF-*α* stimulation, expression of these procancer cytokines/growth factors was found to increase in a dose-dependent manner (Figure 5(a)). Within 24 hours, BMSCs with 20 ng/ml TGF-*α* stimulation produced significantly higher levels of IL-6 (*P* < 0.05), VEGF (*P* < 0.05), FGF10 (*P* < 0.05), FGF17 (*P* < 0.05), and TGF-*β*1 (*P* < 0.05), compared with BMSCs with 0 or 10 ng/ml TGF-*α* treatment (Figure 5(b)). These cytokines are expressed at higher levels in the nontumor tissues of both BMBC and Non-BM-BC while they were seldom found in the benign control (*P* < 0.01) (Figures 5(c) and 5(d)). The level in the nontumor tissues of BMBC is higher than that of the Non-BM-BC group (*P* < 0.05) (Figures 5(c) and 5(d)).

## 4. Discussions

Breast cancer bone metastasis in the early stage of primary lesion is threatening the patients at the rate of 20%. The BMBC patients with removable bone metastasis like the 4 cases in the study were rare. Most of them developed unresectable multiple bone metastases [21–23]. While the interaction between BC cells and BMSCs has been reported to contribute to tumor angiogenesis, invasion, and thus metastasis [24, 25]; the signals (such as cytokines/growth factors) responsible for or underlying this interaction remain largely unclear. In the current study, our results showed that, in breast cancer bone metastasis, TGF-*α* might play a signaling role bridging BC cells and BMSCs and promote the bone metastasis.

In the human BMBC, TGF-*α* was found to be expressed in the bone metastasis lesion, at a higher level than that in primary lesion of BMBC and Non-BM-BC (Figure 2), and TGF-*α* level in primary lesion of BMBC and Non-BM-BC was found to be higher than that in Benign control. These findings suggest that TGF-*α* might be a special signal for breast cancer and its bone metastasis. In the malignant lesion, cancer cells could be the source of TGF-*α*. In this study, TGF-*α* was found to promote BMSCs to increase their transcription of IL-6, VEGF, FGF10, FGF 17, and TGF-*β*1 that could promote BC metastasis obviously (Figure 5). IL-6, VEGF, and TGF-*β*1 have been proved to be commonly overexpressed in human breast cancer cases [26–28], FGF10 played the important role in type III epithelial-mesenchymal transition (EMT) on breast cancer cells and initiation of metastasis [29], and high expression of FGF17 causes tamoxifen resistance [30]. It could be inferred that overexpressed TGF-*α* in BC is an important signal that may bridge bone metastasis, BC, and BMSCs.

Interestingly, in the human BMBC samples, TGF-*α* was also overexpressed in the nontumor tissue around the primary lesion when compared to those in benign control, but it was not expressed at a higher level than that of the nontumor tissue of Non-BM-BC (Figure 3). Our analyses on TGF-*α* expression in the “soil” of BC suggest that TGF-*α* in the microenvironment might not be single signal in breast cancer bone metastasis. However, TGF-*α* in the microenvironment might play important roles in primary breast cancer development, and thus TGF-*α* in normal breast tissue might be a prognostic marker for predicting the risk of BC.

**Figure 6:**
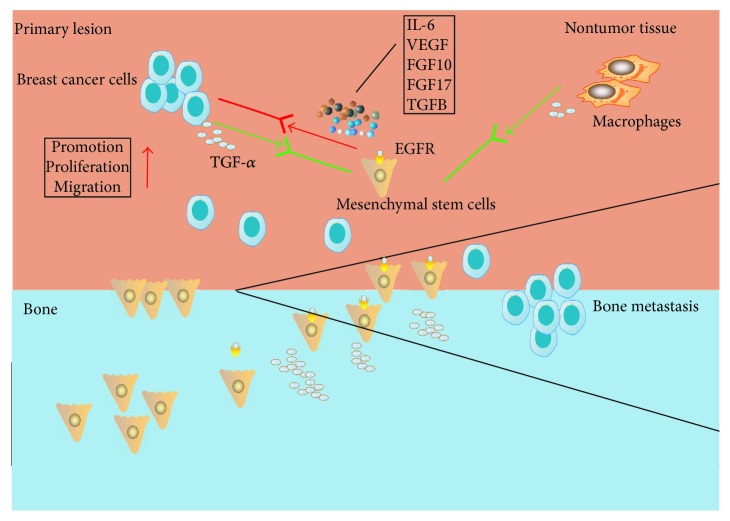
The hypothesis that TGF-*α* stimulates bone marrow-derived mesenchymal stem cells (BMSCs) in breast cancer bone metastasis. Breast cancer influences BMSCs to promote BC metastasis to bone. In the process, breast cancer cells and nontumor tissue macrophages produce TGF-*α* to stimulate BMSCs that express the receptor EGFR, and activated BMSCs produce procancer cytokines/growth factors to promote BC.

In the breast microenvironment, macrophages are an important component and known as a major cellular source of TGF-*α* [31, 32]. In the current study, macrophages were found at higher numbers in the nontumor tissue of BC than benign control (Figure 4). This could potentially explain the higher level of TGF-*α* in the nontumor tissue of BC. In the physiological situation, the interaction of macrophages and BMSCs has been recognized to be important in tissue regeneration during tissue repair, and TGF-*α* is the signal for interaction between macrophages and BMSCs in wound healing [33–36]. Bone metastasis frequently happens to the human loaded bones like the cases presented in the study, and many risk factors could cause damage and subsequent repair in the human loaded bones [37, 38]. With the above known roles of TGF-*α* and our finding of overexpressed TGF-*α* being found mostly in the bone metastasis lesion in the study (Figure 2), it is possible that BC with elevated expression of TGF-*α* might mimic the macrophages in tissue regeneration to influence BMSCs to promote BC to metastasis (Figure 6). On the other hand, as the source of TGF-*α*, macrophages in the nontumor tissue of BC may play a more procancer role than an anticancer role.

In summary, TGF-*α* in BMBC lesion was found to be overexpressed and could be an important signal. TGF-*α* in BC lesion and from nontumor tissue could stimulate BMSCs to promote cancer metastasis. In the further study, more operable BMBC cases would be enrolled. Protein analyses would be performed to define the precise role of TGF-*α* in breast cancer secretion. BMSCs transfected by no-load virus red fluorescent protein would be used in* in vivo* models to evaluate the action mechanism of TGF-*α* locally expressed in BC lesion in influencing the BMSCs and in promoting bone metastasis, information from which could lead to therapeutic targets by controlling TGF-*α* signaling in both primary lesion and the surrounding microenvironment.

## Figures and Tables

**Table 1:** Clinical data of the four bone metastasis breast cancer cases.

	Case 1	Case 2	Case 3	Case 4
Gender	Female	Female	Female	Female
Age	46	66	71	46
Pathology subtypes	Invasive ductal carcinoma	Invasive ductal carcinoma	Invasive lobular carcinoma	Invasive ductal carcinoma
Pathological grade	III	III	III	III
Molecular subtypes	Luminal B	Luminal B	Luminal B	Luminal B
TNM stage, tumor max diameter (cm)	IV, T: 2.2	IV, T: 2.5	IV, T: 3.3	IV, T: 3.7
Location of metastasis	Acetabular bone; axillary lymph node	Thighbone	Humerus; femur; liver; axillary lymph node	Blade bone; thighbone; axillary lymph node
Remote metastasis organ number	One	One	Three	Two
